# Epidemiology and Antibiotic Resistance Patterns of Klebsiella pneumoniae Infections Among Female Patients of a Long-Term Care Hospital in Saudi Arabia

**DOI:** 10.7759/cureus.80008

**Published:** 2025-03-04

**Authors:** Badriah A Alfaifi, Saleh A AlKhaldi, Kholoud A AlHomoud, Wadi A Shuraim

**Affiliations:** 1 Infection Control, Long Term Care Hospital, Riyadh, SAU; 2 Research Center, King Saud Medical City, Riyadh, SAU

**Keywords:** antibiotic resistance, carbapenem-resistant enterobacterales, extended-spectrum beta-lactamases, klebsiella pneumoniae, long-term care, saudi arabia

## Abstract

Background: *Klebsiella pneumoniae* is a gram-negative bacterium naturally found in the gastrointestinal and oropharyngeal tracts but can become an opportunistic pathogen, causing severe healthcare-associated infections, especially in immunocompromised patients. This study aimed to investigate the epidemiology and antibiotic resistance patterns of *K. pneumoniae* infections among female patients in a long-term care hospital in Riyadh, Saudi Arabia.

Methods: A cross-sectional retrospective study examined microbial cultures of 27 female patients with confirmed positive cultures of *K. pneumoniae* who were admitted to a long-term care hospital in Riyadh. Demographic information, in addition to culture sites and antibiotic susceptibility tests, was collected. The associations between patient characteristics and resistance patterns were investigated, focusing on the prevalence of carbapenem-resistant *Enterobacterales* (CRE) and the production of extended-spectrum beta-lactamases (ESBL).

Results: A total of 51.9% (n = 14) of CRE *K.* *pneumoniae* isolates were reported, indicating substantial antibiotic resistance. Patients with bedsores exhibited a lower CRE infection proportion (18.5%) compared to those without bedsores (33.3%), with no statistically significant difference (p = 0.888). Older age (>50 years) demonstrated a higher CRE infection prevalence (29.6%) compared to younger patients (22.2%), with no statistical significance (p = 0.351). Further, all patients reported the widespread use of Foley catheters, and tracheostomy or hospital stay duration did not significantly correlate with resistance patterns. Notably, imipenem exhibited the highest susceptibility rate (66.7%), whereas cotrimoxazole (51.9%) and ampicillin (33.3%) demonstrated a high resistance.

Conclusion: This study highlights the increasing prevalence of CRE and ESBL-producing *K. pneumoniae* infections among female long-term care patients, underscoring the need for enhanced infection control strategies and antimicrobial stewardship programs to mitigate the spread of multidrug-resistant (MDR) pathogens. Targeted interventions, particularly for high-risk patients, are essential for reducing antimicrobial resistance in long-term care settings. Future research should include larger cohorts to further investigate gender-based differences in *K. pneumoniae* resistance patterns.

## Introduction

*Klebsiella pneumoniae* is normally found in the human gastrointestinal and oropharyngeal tracts as part of the natural flora [1,2]. Nonetheless, it can become pathogenic and cause severe infections in other tissues under certain conditions [3]. This bacterium is a major contributor to healthcare-associated infections, especially due to its increasing antibiotic resistance. As a gram-negative bacterium, it is one of the leading causes of bloodstream infections (BSIs), second only to *Escherichia coli*. BSIs can arise as primary infections without a clear source but are often associated with the spread of pathogens from known infection sites such as the urinary tract, gastrointestinal system, intravenous or urinary catheters, and respiratory sites [4,5].

Certain health conditions increase the risk of *K. pneumoniae* infections. These include diabetes, liver diseases, and cancer, which together make up around 50% of community-acquired BSIs caused by *K. pneumoniae* [6,7]. Chronic lung disease is also a significant risk factor, especially for elderly patients. Other factors that contribute to the risk are previous antibiotic use and invasive medical procedures like endotracheal intubation, bladder catheterization, and intravenous catheterization [8]. Moreover, hypervirulent strains of *K. pneumoniae* can lead to severe, widespread infections such as liver and prostate abscesses, pneumonia, meningitis, and endophthalmitis [9].

Over the past two decades, *K. pneumoniae* has garnered increasing attention due to its growing antibiotic resistance and the severe health problems it causes [9,10]. This bacterium is now a major cause of hospital-acquired pneumonia, responsible for about 10% of all such infections, and is the second most prevalent gram-negative bacterium in hospital environments [11,12]. Multidrug-resistant (MDR) strains of *K. pneumoniae* are particularly concerning, contributing to various healthcare-associated infections like pneumonia, urinary tract infections (UTIs), and BSIs. These MDR strains produce extended-spectrum beta-lactamases (ESBL) and carbapenemases, resulting in higher mortality rates, especially among immunocompromised and critically ill patients [13,14]. Additionally, the rise of extensively drug-resistant (XDR) and pan-drug-resistant strains poses an even greater challenge, as these strains are resistant to nearly all available antimicrobial agents [9,15].

Furthermore, epidemiological studies reveal a global spread of hypervirulent and MDR *K. pneumoniae* strains, particularly in Southeast Asia, with notable clinical impacts. These strains are associated with severe infections such as pneumonia, UTIs, and BSIs, which are increasingly resistant to last-resort antibiotics like carbapenems. This alarming trend underscores the urgent need for effective infection control measures and antimicrobial stewardship programs, especially in long-term care facilities where patients are at greater risk [16].

Long-term care hospitals accommodate many frail patients with chronic health conditions, making them highly susceptible to infections [17]. A longitudinal study revealed that patients colonized with *K. pneumoniae* had a higher likelihood of developing respiratory infections, UTIs, or BSIs. Specifically, 5.2% of colonized patients developed subsequent infections, compared to just 1.3% of non-colonized patients. These findings highlight the critical importance of monitoring colonization in high-risk healthcare environments [18,19].

The rising prevalence of MDR *K. pneumoniae* infections has prompted the World Health Organization (WHO) to designate this pathogen as critical because of its public health impact [20]. National research mirrors these global concerns, showing that MDR *K. pneumoniae* strains are becoming increasingly common in healthcare environments [21,22]. A recent study from Saudi Arabia published last year highlighted a significant occurrence of MDR *K. pneumoniae* infections in long-term care facilities, particularly affecting elderly male patients and those with bedsores [23].

The aim of this study is to examine the prevalence of infections, associated risk factors, antimicrobial resistance profiles, and treatment outcomes in a long-term care facility in Saudi Arabia. In addition, the findings aim to provide evidence-based recommendations for targeted interventions and policies to mitigate the spread of *K. pneumoniae* and enhance patient care within this facility.

Study objectives

(i) To investigate the epidemiology of *K. pneumoniae* infections in a female ward at Riyadh's Long Term Care Hospital, Saudi Arabia. (ii) To determine the antibiotic resistance patterns of MDR *K. pneumoniae* infections in female subjects hospitalized in the facility.

## Materials and methods

This cross-sectional retrospective study investigated *K. pneumoniae* microbial culture data of female subjects at a long-term care hospital in Riyadh, Saudi Arabia. We recruited subjects with confirmed, positive *K. pneumoniae* cultures collected from June 2024 to November 2024. Inclusion criteria consisted of female patients hospitalized at the facility at that time with confirmed *K. pneumoniae* infections based on positive microbial culture results. Outpatients and long-term care patients with non-pathogenic *K. pneumoniae* colonization were excluded.

Data were collected from microbiological records maintained by the Infection Control Department, ensuring the accuracy and quality of the collected information. Extracted data included patient demographics, culture sites (blood, sputum, urine, wounds), antimicrobial susceptibility results, clinical risk factors (e.g., bedsores and tracheostomy), and 30-day post-diagnosis mortality associated with carbapenem-resistant Enterobacterales (CRE). A total of 27 samples met the inclusion criteria.

CRE isolates were identified through a two-step process: disc diffusion testing based on the Clinical and Laboratory Standards Institute (CLSI) guidelines [24], followed by minimum inhibitory concentration (MIC) testing using the VITEK 2 automated system (bioMérieux, Marcy-l'Étoile, France) for confirmation [25]. Extended-spectrum beta-lactamase (ESBL) production was determined using the double-disc synergy test per the CLSI recommendations [26]. Antibiotic susceptibility testing (AST) followed the Kirby-Bauer disc diffusion method [27], evaluating resistance against beta-lactams (ampicillin, Augmentin, cefotaxime, cefepime, ceftriaxone, ceftazidime), carbapenems (imipenem, meropenem, ertapenem), aminoglycosides (amikacin, gentamicin), fluoroquinolones (ciprofloxacin), polymyxins (colistin), tetracyclines (tigecycline), and sulfonamides (cotrimoxazole). Zone diameters were measured with digital calipers and interpreted according to the CLSI breakpoints [24].

To ensure test reliability, quality control measures included the use of positive and negative control strains (*K. pneumoniae* ATCC 700603 for ESBL, ATCC BAA-1705 for CRE, and *E. coli* ATCC 25922 as a negative control). Daily calibration of equipment and blinded duplicate testing of 10% of isolates ensured accuracy, with additional re-testing of randomly selected samples for consistency [28].

The study obtained ethical approval from the Institutional Review Board, King Saud Medical City, Ministry of Health, Saudi Arabia (# H1RI-05-Aug24-01), adhering to the principles outlined in the Declaration of Helsinki, current legislation on clinical research, and Good Clinical Practice guidelines. All patient information was kept confidential throughout the study, and data protection was assured.

The data were analyzed using SPSS software for Windows, version 24 (IBM Corp., Armonk, NY). Descriptive statistical analysis was performed, with characterized patient demographics, culture site distribution, and antibiotic resistance patterns. Chi-square tests explored potential associations among medical factors, CRE prevalence, and ESBL resistance patterns in *K. pneumoniae* infections. A p-value <0.05 was considered statistically significant.

## Results

A total of 27 female patients with a mean age of 52.37 years (±13.58) generated 27 samples. The average hospital stay duration was 509.52 days (±988.21 days). Among the patients, 37.0% experienced bedsores, and all had Foley catheters. Additionally, 59.3% received a tracheostomy. We obtained specimen cultures from blood (14.8%), sputum (18.5%), urine (33.3%), and wounds (33.3%). We revealed no deaths within 30 days after detecting CRE. Additionally, 51.9% of the patients were resistant to CRE and 48.1% to ESBL (Table 1).

**Table 1:** Clinical characteristics of the patients with Klebsiella pneumoniae (n = 27). This table presents a comprehensive overview of the characteristics of female patients with *K. pneumoniae* infections in a long-term care hospital in Riyadh, Saudi Arabia. CRE: carbapenem resistance; ESBL: extended-spectrum beta-lactamase.

Characteristics	Frequency	Percent
Age		
From 20-50 years	14	51.9%
Over 50 years	13	48.1%
Hospital stay duration		
Under 200 days	14	51.9%
Over 200 days	13	48.1%
Bedsore(s)		
Yes	10	37.0%
No	17	63.0%
Foley catheter		
Yes	27	100.0%
Tracheostomy		
Yes	16	59.3%
No	11	40.7%
Culture site		
Blood	4	14.8%
Sputum	5	18.5%
Urine	9	33.3%
Wound	9	33.3%
Antibiotic resistance		
CRE	14	51.9%
ESBL	13	48.1%
Death within 30 days		
No	27	100.0%

Antimicrobial susceptibility analysis of the *K. pneumoniae* isolates revealed varying resistance levels across different antibiotics. We observed high resistance levels for cotrimoxazole (51.9%), ampicillin (33.3%), and imipenem (22.2%). Intermediate susceptibility was rare, with only meropenem (7.4%) and cefotaxime (3.7%) demonstrating intermediate resistance levels. Several antibiotics showed notable susceptibility. Imipenem exhibited the highest susceptibility rate at 66.7%, making it a potential treatment option. Additionally, ertapenem demonstrated substantial susceptibility at 55.6%. Moreover, amikacin (48.1%) and cotrimoxazole (44.4%) demonstrated significant susceptibility levels.

Other antibiotics with lower resistance levels included augmentin (7.4% resistance, 22.2% susceptibility), cefotaxime (18.5% resistance, 33.3% susceptibility), and gentamicin (14.8% resistance, 37.0% susceptibility). Interestingly, some antibiotics, such as cefepime, ciprofloxacin, colistin, and tigecycline, demonstrated no resistance among the isolates tested. Overall, the data indicate that imipenem, ertapenem, and amikacin could be effective options for treating *K. pneumoniae* infections in this population, considering their higher susceptibility rates. However, the presence of high resistance rates for predominant antibiotics, such as cotrimoxazole and ampicillin, indicates the need for careful selection of antimicrobial therapy based on susceptibility testing (Table 2).

**Table 2:** Antimicrobial susceptibility testing for Klebsiella pneumoniae (n = 27). This table provides a detailed breakdown of the antimicrobial susceptibility testing results for the 27 *K. pneumoniae* isolates obtained from the study participants. Antibiotics are classified based on their type, and the percentage (%) of resistance, intermediate susceptibility, and susceptibility are determined for each antibiotic.

Type	Resistant	Intermediate	Sensitive
Ampicillin	9 (33.3%)	-	-
Augmentin	2 (7.4%)	-	6 (22.2%)
Amikacin	4 (14.8%)	-	13 (48.1%)
Cefepime	-	-	1 (3.7%)
Cefotaxime	5 (18.5%)	1 (3.7%)	9 (33.3%)
Ceftazidime	2 (7.4%)	-	1 (3.7%)
Ceftriaxone	2 (7.4%)	-	4 (14.8%)
Ciprofloxacin	-	-	-
Cotrimoxazole	14 (51.9%)	-	12 (44.4%)
Gentamicin	4 (14.8%)	-	10 (37.0%)
Imipenem	6 (22.2%)	-	18 (66.7%)
Meropenem	4 (14.8%)	2 (7.4%)	11 (40.7%)
Ertapenem	5 (18.5%)	-	15 (55.6%)
Tazocin	-	-	4 (14.8%)
Colistin	-	-	-
Tigecycline	-	-	-
Nitrofurantoin	4 (14.8%)	-	8 (29.6%)

No significant association was found between bedsores and CRE or ESBL resistance patterns (p = 0.888). Patients with bedsores demonstrated similar proportions of CRE (18.5%) and ESBL (18.5%) infections compared to those without bedsores, who showed slightly higher CRE (33.3%) and ESBL (29.6%) infection rates. Therefore, bedsores do not appear to significantly affect the likelihood of developing CRE or ESBL infections in this patient population. All participants had Foley catheters, a well-known risk factor for healthcare-associated infections. The prevalence of CRE and ESBL among patients with Foley catheters was high, with 51.9% demonstrating CRE and 48.1% showing ESBL. However, the widespread use of Foley catheters among the study population precluded statistical analysis of their association with resistance patterns. Moreover, a tracheostomy was also not significantly associated with CRE or ESBL infections (p = 0.825). Patients with a tracheostomy demonstrated similar proportions of CRE (29.6%) and ESBL (29.6%) infections compared to those without a tracheostomy, who showed slightly lower rates of CRE (22.2%) and ESBL (18.5%) infections (Table 3).

**Table 3:** Medical and other factors associated with CRE and ESBL resistance patterns in female patients with Klebsiella pneumoniae infections. This table illustrates the associations between *K. pneumoniae* infections, CRE prevalence, and ESBL resistance patterns in female patients, highlighting potential risk factors for multidrug-resistant (MDR) strains. P-values are based on chi-square tests. * Significant when the alpha criterion for p-value is set at 0.05. A p-value <0.05 indicates statistical significance. For "Foley catheter," the chi-square test was not applicable due to the constant nature of the variable (100% of cases). CRE: carbapenem resistance; ESBL: extended-spectrum beta-lactamase.

Items	Characteristic	CRE	ESBL	Total	P-value
Bedsores	Yes	5 (18.5%)	5 (18.5%)	10 (37.0%)	0.883
	No	9 (33.3%)	8 (29.6%)	17 (63.0%)	
Foley catheter	Yes	14 (51.9%)	13 (48.1%)	27 (100.0%)	N/A
Tracheostomy	Yes	8 (29.6%)	8 (29.6%)	16 (59.3%)	0.816
	No	6 (22.2%)	5 (18.5%)	11 (40.7%)	
Culture site	Blood	3 (11.1%)	1 (3.7%)	4 (14.8%)	0.367
	Sputum	2 (7.4%)	3 (11.1%)	5 (18.5%)	
	Urine	6 (22.2%)	3 (11.1%)	9 (33.3%)	
	Wound	3 (11.1%)	6 (22.2%)	9 (33.3%)	
Age categories	20-50 years	6 (22.2%)	8 (29.6%)	14 (51.9%)	0.332
	Over 50 years	8 (29.6%)	5 (18.5%)	13 (48.1%)	
Days of stay	Under 200 days	6 (22.2%)	8 (29.6%)	14 (51.9%)	0.332
	Over 200 days	8 (29.6%)	5 (18.5%)	13 (48.1%)	

The distribution of resistance patterns differed by culture site. CRE was more prevalent in urine cultures (22.2%), whereas ESBL-producing isolates were most predominant in wound cultures (22.2%). Other culture sites, including blood and sputum, demonstrated lower prevalence rates for both CRE and ESBL. However, the association between culture site and resistance patterns was not statistically significant (p = 0.298). Age was not significantly related to resistance patterns (p = 0.351). Patients aged <50 years had slightly higher ESBL (29.6%) rates compared to CRE (22.2%), whereas patients aged ≥50 years demonstrated higher CRE (29.6%) and lower ESBL rates (18.5%).

Hospital stay duration demonstrated no significant association with CRE or ESBL resistance patterns (p = 0.294). Patients with a hospital stay of <360 days exhibited slightly higher rates of ESBL (37.0%) compared to CRE (29.6%). Conversely, patients with a hospital stay of >360 days demonstrated higher CRE (22.2%) and lower ESBL rates (11.1%). Thus, hospital stay duration does not appear to significantly affect the prevalence of CRE or ESBL infections in this patient population.

## Discussion

This study investigated the epidemiological characteristics and antibiotic resistance patterns of *Klebsiella pneumoniae* infections among female patients hospitalized in a long-term care hospital in Riyadh, Saudi Arabia. We also aimed to assess infection prevalence, associated risk factors, and the antibiotic resistance profiles of *K. pneumoniae* in this setting.

The study’s main findings revealed a high prevalence (>50%) of CRE among the patients, with 51.9% of isolates resistant to carbapenems. Additionally, 48.1% of the isolates were ESBL-producing *K. pneumoniae*. This is a concerning trend, as CRE infections significantly limit treatment options and are associated with high mortality rates, particularly among critically ill and immunocompromised patients (Table 1). However, despite the high CRE rate, no significant epidemiological transition was observed in the pathogen profile or within the study population over time.

We also evaluated potential risk factors for antimicrobial resistance in this patient population. The results showed that bedsores were present in 37.0% of patients, yet there was no significant association between bedsores and CRE or ESBL resistance (p = 0.883). Patients with bedsores had 18.5% CRE and 18.5% ESBL infections, while those without bedsores exhibited 33.3% CRE and 29.6% ESBL infections. Unlike in our prior study on male patients, where bedsores were significantly linked to CRE infections (p = 0.034), the current findings suggest that bedsores did not significantly contribute to CRE development among female patients [23]. This may indicate a difference in the underlying risk factors influencing antibiotic resistance between genders.

Another potential risk factor evaluated was tracheostomy, which was present in 59.3% of patients. However, no significant association was found between tracheostomy and antimicrobial resistance (p = 0.816). Among patients with a tracheostomy, 29.6% had CRE and 29.6% had ESBL infections, while patients without a tracheostomy exhibited 22.2% CRE and 18.5% ESBL infections. Although tracheostomy patients showed a slightly higher prevalence of resistance, this association was not statistically significant. Given that tracheostomy is often required for patients with prolonged mechanical ventilation or chronic respiratory conditions, these patients may have higher exposure to hospital-acquired infections, potentially contributing to elevated antimicrobial resistance rates. However, further studies with larger sample sizes are needed to determine whether tracheostomy plays a role in antimicrobial resistance in long-term care settings.

Regarding culture site distribution, CRE was most frequently isolated from urine cultures (22.2%), whereas ESBL-producing isolates were most prevalent in wound cultures (22.2%). Blood (11.1% CRE, 3.7% ESBL) and sputum (7.4% CRE, 11.1% ESBL) cultures had lower prevalence rates for both resistance types. However, there was no statistically significant association between culture site and resistance patterns (p = 0.367).

We also examined age as a potential factor influencing resistance patterns, but the results showed no significant association (p = 0.332). Patients aged 20-50 years had higher ESBL rates (29.6%) compared to CRE (22.2%), whereas patients over 50 years exhibited higher CRE rates (29.6%) and lower ESBL rates (18.5%). This age-related variation in resistance patterns has been observed in previous studies, with older patients often experiencing prolonged hospital stays and increased antibiotic exposure, potentially predisposing them to CRE infections [22]. However, in contrast to our male patient study, where older males had a significantly higher prevalence of CRE (34.5% vs. 17.2% in younger patients, p = 0.069), age did not appear to be a major determinant of resistance in the female cohort [23].

Hospital stay duration also showed no significant association with antimicrobial resistance (p = 0.332). Patients hospitalized for less than 200 days had higher ESBL prevalence (29.6%) compared to CRE (22.2%), whereas those hospitalized for over 200 days exhibited higher CRE prevalence (29.6%) and lower ESBL rates (18.5%). These findings suggest that prolonged hospitalization may be linked to an increased risk of CRE infections, possibly due to longer exposure to antibiotics and hospital-acquired pathogens. However, the lack of statistical significance may indicate that additional risk factors, such as underlying comorbidities or prior antibiotic use, play a more substantial role in determining antimicrobial resistance patterns.

The high prevalence (>50%) of CRE among *K. pneumoniae* isolates in this study is consistent with previous reports from Saudi Arabia. A study conducted in King Fahd Medical City, Riyadh, found that 36.1% of *K. pneumoniae* isolates from blood cultures were CRE-positive [22]. Additionally, another study reported a significant rise in carbapenem resistance rates, from 6.6% for imipenem in 2011 to 59.9% in 2021, in a tertiary care hospital in Makkah, Saudi Arabia [24]. These findings underscore the critical challenge of antimicrobial resistance in healthcare facilities and highlight the urgent need for enhanced infection control strategies.

Our study results also align with global trends in MDR *K. pneumoniae* infections, where CRE has emerged as a major public health threat. The WHO has classified carbapenem-resistant Enterobacteriaceae as a priority pathogen, emphasizing the need for novel antimicrobial therapies and improved infection prevention measures [20].

While this study provides valuable insights into the resistance patterns of *K. pneumoniae* infections among female patients, several limitations must be acknowledged. The study was conducted in a single hospital with a relatively small sample size (n = 27), which may limit the generalizability of the findings. Additionally, all patients in the study had Foley catheters, preventing an assessment of their potential contribution to antimicrobial resistance. The low patient turnover rate and limited bed capacity at the facility further constrained patient recruitment, potentially affecting the statistical power of some analyses.

## Conclusions

To conclude, the results highlight the increasing prevalence and spread of MDR *K. pneumoniae*, especially CRE, in healthcare facilities. Risk factors, such as the ubiquitous presence of Foley catheters, older age, and high MDR rates, emphasize the urgent need for strategies that comprehensively address these challenging healthcare-associated infections. Epidemiological surveillance studies of *K. pneumoniae* in both genders are warranted. The results must be compared with existing data to provide the required recommendations for the safe use of antibiotics.

## References

[1] Assoni L, Couto AJ, Vieira B, Milani B, Lima AS, Converso TR, Darrieux M (2024). Animal models of Klebsiella pneumoniae mucosal infections. Front Microbiol.

[2] Chen Q, Wang M, Han M, Xu L, Zhang H (2023). Molecular basis of Klebsiella pneumoniae colonization in host. Microb Pathog.

[3] Lisowska-Łysiak K, Lauterbach R, Międzobrodzki J, Kosecka-Strojek M (2021). Epidemiology and pathogenesis of Staphylococcus bloodstream infections in humans: a review. Pol J Microbiol.

[4] Murray CJ, Ikuta KS, Sharara F (2022). Global burden of bacterial antimicrobial resistance in 2019: a systematic analysis. Lancet.

[5] Ko WC, Paterson DL, Sagnimeni AJ (2002). Community-acquired Klebsiella pneumoniae bacteremia: global differences in clinical patterns. Emerg Infect Dis.

[6] Lee TC, Arif AM, Razali NH (2023). Clinical characteristics and outcomes of Klebsiella pneumonia bacteraemia in adult. IIUM Med J Malays.

[7] Lin YT, Jeng YY, Chen TL, Fung CP (2010). Bacteremic community-acquired pneumonia due to Klebsiella pneumoniae: clinical and microbiological characteristics in Taiwan, 2001-2008. BMC Infect Dis.

[8] Navon-Venezia S, Kondratyeva K, Carattoli A (2017). Klebsiella pneumoniae: a major worldwide source and shuttle for antibiotic resistance. FEMS Microbiol Rev.

[9] Lima AM, de Melo ME, Alves LC, Brayner FA, Lopes AC (2014). Investigation of class 1 integrons in Klebsiella pneumoniae clinical and microbiota isolates belonging to different phylogenetic groups in Recife, State of Pernambuco. Rev Soc Bras Med Trop.

[10] Abdelsalam MF, Abdalla MS, El-Abhar HS (2018). Prospective, comparative clinical study between high-dose colistin monotherapy and colistin-meropenem combination therapy for treatment of hospital-acquired pneumonia and ventilator-associated pneumonia caused by multidrug-resistant Klebsiella pneumoniae. J Glob Antimicrob Resist.

[11] Mohd Asri NA, Ahmad S, Mohamud R (2021). Global prevalence of nosocomial multidrug-resistant Klebsiella pneumoniae: a systematic review and meta-analysis. Antibiotics (Basel).

[12] Di Tella D, Tamburro M, Guerrizio G, Fanelli I, Sammarco ML, Ripabelli G (2019). Molecular epidemiological insights into colistin-resistant and carbapenemases-producing clinical Klebsiella pneumoniae isolates. Infect Drug Resist.

[13] Martin RM, Bachman MA (2018). Colonization, infection, and the accessory genome of Klebsiella pneumoniae. Front Cell Infect Microbiol.

[14] Sid Ahmed MA, Hamid JM, Hassan AM, Abu Jarir S, Bashir Ibrahim E, Abdel Hadi H (2024). Phenotypic and genotypic characterization of pan-drug-resistant Klebsiella pneumoniae isolated in Qatar. Antibiotics (Basel).

[15] Salawudeen A, Raji YE, Jibo GG (2023). Epidemiology of multidrug-resistant Klebsiella pneumoniae infection in clinical setting in South-Eastern Asia: a systematic review and meta-analysis. Antimicrob Resist Infect Control.

[16] Chen HY, Jean SS, Lee YL, Lu MC, Ko WC, Liu PY, Hsueh PR (2021). Carbapenem-resistant Enterobacterales in long-term care facilities: a global and narrative review. Front Cell Infect Microbiol.

[17] Dorman MJ, Short FL (2017). Genome watch: Klebsiella pneumoniae: when a colonizer turns bad. Nat Rev Microbiol.

[18] Chang D, Sharma L, Dela Cruz CS, Zhang D (2021). Clinical epidemiology, risk factors, and control strategies of Klebsiella pneumoniae infection. Front Microbiol.

[19] Martin MJ, Corey BW, Sannio F (2021). Anatomy of an extensively drug-resistant Klebsiella pneumoniae outbreak in Tuscany, Italy. Proc Natl Acad Sci U S A.

[20] Al Bshabshe A, Al-Hakami A, Alshehri B (2020). Rising Klebsiella pneumoniae infections and its expanding drug resistance in the intensive care unit of a tertiary healthcare hospital, Saudi Arabia. Cureus.

[21] Al-Zalabani A, AlThobyane OA, Alshehri AH, Alrehaili AO, Namankani MO, Aljafri OH (2020). Prevalence of Klebsiella pneumoniae antibiotic resistance in Medina, Saudi Arabia, 2014-2018. Cureus.

[22] Hafiz TA, Alanazi S, Alghamdi SS (2023). Klebsiella pneumoniae bacteraemia epidemiology: resistance profiles and clinical outcome of King Fahad Medical City isolates, Riyadh, Saudi Arabia. BMC Infect Dis.

[23] Alfaifi BA, Alkhaldi SA, Alanazi MD, Shuraim WA, Aldossry MA, Alzahrani HS, Alhaitei FA (2024). Epidemiology and antibiotic resistance patterns of Klebsiella pneumoniae infections among male patients in a long-term care hospital in Riyadh, Saudi Arabia: a retrospective study. Cureus.

[24] Jalal NA, Al-Ghamdi AM, Momenah AM (2023). Prevalence and antibiogram pattern of Klebsiella pneumoniae in a tertiary care hospital in Makkah, Saudi Arabia: an 11-year experience. Antibiotics (Basel).

[25] Funke G, Monnet D, deBernardis C, von Graevenitz A, Freney J (1998). Evaluation of the VITEK 2 system for rapid identification of medically relevant gram-negative rods. J Clin Microbiol.

[26] Jarlier V, Nicolas MH, Fournier G, Philippon A (1988). Extended broad-spectrum β-lactamases conferring transferable resistance to newer β-lactam agents in Enterobacteriaceae: hospital prevalence and susceptibility patterns. Rev Infect Dis.

[27] Bauer AW, Kirby WM, Sherris JC, Turck M (1966). Antibiotic susceptibility testing by a standardized single disk method. Am J Clin Pathol.

[28] Hall SL (2024). Evaluating the contribution of the frmRAB operon on the in vitro activity of chlorine-based disinfectants among KPC-producing carbapenem-resistant Enterobacterales. https://jscholarship.library.jhu.edu/items/f495ad6c-645a-4380-8b4e-1738164ebdce.

